# LMDIPred: A web-server for prediction of linear peptide sequences binding to SH3, WW and PDZ domains

**DOI:** 10.1371/journal.pone.0200430

**Published:** 2018-07-12

**Authors:** Debasree Sarkar, Tanmoy Jana, Sudipto Saha

**Affiliations:** Bioinformatics Centre, Bose Institute, Kolkata, India; Universita degli Studi di Roma Tor Vergata, ITALY

## Abstract

Protein-peptide interactions form an important subset of the total protein interaction network in the cell and play key roles in signaling and regulatory networks, and in major biological processes like cellular localization, protein degradation, and immune response. In this work, we have described the LMDIPred web server, an online resource for generalized prediction of linear peptide sequences that may bind to three most prevalent and well-studied peptide recognition modules (PRMs)—SH3, WW and PDZ. We have developed support vector machine (SVM)-based prediction models that achieved maximum Matthews Correlation Coefficient (MCC) of 0.85 with an accuracy of 94.55% for SH3, MCC of 0.90 with an accuracy of 95.82% for WW, and MCC of 0.83 with an accuracy of 92.29% for PDZ binding peptides. LMDIPred output combines predictions from these SVM models with predictions using Position-Specific Scoring Matrices (PSSMs) and string-matching methods using known domain-binding motif instances and regular expressions. All of these methods were evaluated using a five-fold cross-validation technique on both balanced and unbalanced datasets, and also validated on independent datasets. LMDIPred aims to provide a preliminary bioinformatics platform for sequence-based prediction of probable binding sites for SH3, WW or PDZ domains.

## Introduction

Protein-protein interactions (PPIs) are primary regulators of protein functions [1], and a large number of PPIs are known to be mediated by short contiguous peptide segments, which are represented as sequence patterns known as Linear Motifs (LMs) [2]. LM peptides are generally found in intrinsically disordered regions, and act as recognition sites for low-affinity but highly specific domain-peptide interactions, thus mediating PPIs that are transient, yet critical for various signaling and regulatory pathways [3]. Peptide-mediated PPIs have been implicated in several diseases like cancer and some neurodegenerative and genetic disorders [4]. Hence, identification of such short LM peptide sequences within proteins may be useful in targeting specific disease-associated domain-peptide interactions for therapeutic modulation [5]. But, the computational challenge in predicting the occurrence of such peptides is that these sequences are too short to generate a statistically significant hit that may distinguish them from non-functional sites, especially in large protein sequences. Nevertheless, several generalized computational methods have been devised by various research groups to aid in the discovery of novel peptide motifs [6–8]. The well-known data repositories that compile known LM instances, like the Eukaryotic Linear Motif resource (ELM) [9], Minimotif Miner (MnM) [10], and Scansite [11], also provide web-interfaces for searching the database LMs within query protein sequences. The iSPOT web tool [12] provides a structure-based approach for prediction of peptides that may bind to three specific peptide recognition modules (PRMs) namely, SH3, PDZ and WW. The most recent online resource for prediction of specific domain-peptide interactions is MoDPepInt [13], which comprises of three different tools, i.e. SH2PepInt [14], SH3PepInt [15] and PDZPepInt [16], for predicting the binding partners of SH2, SH3 and PDZ domains, respectively. However, all the existing methods were developed either solely based on regular expression matching or entirely around machine learning algorithms, but none utilized a combination of both these techniques to increase prediction efficiency. Hence, we thought it would be worthwhile to develop an online computational resource for prediction of specific domain binding peptides by integrating both the approaches of machine learning and simple sequence/motif matching to give a better combined prediction result.

We have formulated four different prediction strategies for LM peptides binding to SH3, WW and PDZ domains and assembled them all into a web-based bioinformatics resource named **L**inear **M**otif **D**omain **I**nteraction **Pred**iction (**LMDIPred**). We had previously compiled experimentally validated LM instances from published data into a manually curated database called LMPID (Linear Motif mediated Protein-Protein Interaction Database) [17]. Herein, we observed that the highest number of ligand peptides reported were for SH3, WW and PDZ domains. Proteins containing these domains were known to play crucial roles in critical diseases like cancer and neurodegenerative disorders [18–23], and hence, peptides binding to these three domains were extensively studied. Although prediction servers have been previously developed for SH3, WW and PDZ binding peptides, we selected SH3, WW and PDZ domains for developing better prediction methods for domain-specific peptide interactions. However, validated LM instances specific to each subclass of the above-mentioned domains were not adequate in number for training subclass-specific machine-learning models. We have, therefore, trained our Support Vector Machine (SVM) classifiers on the entire dataset of ligand peptides for each particular domain, thus providing a generalized prediction of domain-binding without considering the domain subtypes. It was observed that majority of the peptides binding to SH3, WW or PDZ domains were 6-mers in length.

SH3 and WW domains bind to proline-rich sequences, but the exact sequence specificities are slightly different for each domain [24–26]. On the other hand, PDZ domains specifically recognize and bind to short C-terminal peptide motifs, but can also recognize internal sequences that structurally mimic a terminus [27]. The respective domain-binding peptide sequences were analyzed to identify the key features of the ligands of each domain that clearly distinguished them from the ligands of the other two domains or random peptide sequences of the same length. After identification of such features, these were used to develop statistical prediction models to predict SH3, WW and PDZ binding peptides with high precision. Besides, we also used simple string-matching algorithms to detect either exact sequence matches to the known binding instances for each domain, or matches to the set of regular expressions describing the LMs binding to each domain, or matches to PSSMs generated from sets of sequences binding to each domain. All these four prediction strategies were incorporated into a publicly available web server which is freely accessible at *http://bicresources.jcbose.ac.in/ssaha4/lmdipred/*.

## Materials and methods

### Creation of datasets

- **Positive dataset-** LMPID Database lists 153, 156 and 274 entries against SH3, WW and PDZ domain respectively. If more than one LMPID entries represent the same motif sequence at the same position of a protein, then only one of these entries was included in the positive dataset. However, if the same sequence came either from a different position of the protein or from another protein or protein isoform, then all such entries were inserted in the positive dataset. Thus, a non-redundant and non-overlapping dataset consisting of 115, 140 and 165 peptide instances binding to SH3, WW and PDZ domains respectively, were extracted from the LMPID database [17], and used as positive training examples for the respective class of peptides. We wanted to use the same dataset for comparing the four methods and observed that 6-residue long peptides were the most abundant in all the three classes of peptide ligands (**Fig A** in **S1 File**). Furthermore, our preliminary studies with SVM classifier showed that 6-mer peptides produced better prediction results for SH3 and WW and 4-mer peptides for PDZ domain ligands (**Fig B** and **Table D** in **S1 File**). Hence, we decided to use 6-mer peptides as input for SH3 and WW and 4-mer for PDZ binding peptides, during five-fold cross-validation studies.
- **Negative dataset-** A set of 3960 fasta-formatted protein sequences [3192 from Oryza sativa subsp. japonica (short-grained Asian rice), 400 from Solanum tuberosum (potato), and 368 from Triticum aestivum (common wheat)] were downloaded from UniProtKB/Swiss-Prot, the manually annotated section of the UniProt KnowledgeBase [28]. Perl scripts were used to extract a set of 120 peptides from random positions within these sequences, and were used as negative training examples, along with positive examples of the other two classes. Negative dataset consisted of 6-residue long peptides for SH3 and WW domains and 4-residue long peptides for PDZ domain.
- **Training dataset-** The unbalanced training dataset, therefore, consisted of 115 positive and 425 (140+165+120) negative examples (~1:4) for SH3 ligands, 140 positive and 400 (115+165+120) negative examples (~1:3) for WW ligands, and 165 positive and 375 (115+140+120) negative examples (~1:2) for PDZ ligands. Furthermore, we also created balanced (positive: negative = 1:1) datasets for all four methods by including 115 positive and 115 (30+30+55) negative examples for SH3 ligands, 140 positive and 140 (45+45+50) negative examples for WW ligands, and 165 positive and 165 (50+50+65) negative examples for PDZ ligands.
- **Independent or validation datasets-** To validate our proposed methods on independent datasets not used for training or testing, we used 62 experimentally validated PDZ-binding 10-mer mouse peptides from *Stiffler et al* [29], and 25 experimentally validated SH3-binding yeast peptides of variable length from *Tonikian et al* [30].

### Computation of the sequence composition

In the past, compositional features of peptide sequences have been used successfully for developing methods for predicting domain-peptide interactions [15, 31]. In our study also, statistical prediction models have been developed using different compositional features like amino acid, dipeptide and tripeptide composition (AAC, DPC and TPC, respectively), which were calculated using Perl scripts as described below:

#### Amino acid composition (AAC)

Amino acid composition of each input peptide was calculated as the percentage of each amino acid i (where i = 1 to 20) present in the peptide, using the following equation:

$$Composition\;of\;amino\;acid\;i=\frac{Number\;of\;amino\;acid\;i\;in\;the\;peptide}{Total\;number\;of\;amino\;acids\;in\;the\;peptide}\times 100$$

#### Dipeptide composition (DPC)

Dipeptide composition was encoded using a feature length of 20X20 = 400 representing all possible amino acid combinations, thereby encapsulating information about the composition of amino acids as well as their relative ordering in the sequence. The percentage of each dipeptide j (where j = 1 to 400) present in a peptide was calculated using the following equation:

$$Composition\;of\;dipeptide\;j=\frac{Number\;of\;dipeptide\;j\;in\;the\;peptide}{Total\;number\;of\;dipeptides\;in\;the\;peptide}\times 100$$

#### Tripeptide composition (TPC)

Among 8000 possible tripeptides, we found 94 tripeptides occurring in more than one SH3 domain ligands, 87 in more than one WW ligands and 133 in more than one PDZ ligands. We have used the composition of only these over-represented tripeptides within each ligand class as input features during generation of prediction models for the corresponding class, to reduce the dimensions of the input vectors, and thereby improve prediction performance. The tripeptide composition of a particular significant tripeptide k (k = 1 to 94 for SH3, 1 to 87 for WW and 1 to 133 for PDZ binding peptides respectively) was therefore calculated using the following equation:

$$Composition\;of\;tripeptide\;k=\frac{Number\;of\;tripeptide\;k\;in\;the\;peptide}{Total\;number\;of\;tripeptides\;in\;the\;peptide}\times 100$$

#### Location at C-terminal for PDZ ligands

Since PDZ binding peptides are predominantly found at the C-terminus of proteins, the location of the peptide at the C-terminal end of the whole protein sequence was also considered as an additional parameter in the input feature vectors for prediction of PDZ ligands, in addition to the compositional features.

### Support vector machine (SVM)

The SVM-based classification was carried out using the Radial Basis Function (RBF) kernel from the SVM^light^ package Version 6.02 by T. Joachims [32]. Different parameters were optimized to get the best performance on the training datasets (**Table A** in **S1 File)**.

### Construction of Position-Specific Scoring Matrices (PSSMs)

PSSMs for the 6-mer SH3 & WW and 4-mer PDZ ligands were computed from alignments of the 115 SH3-domain binding peptides, 140 WW-domain binding peptides and 165 PDZ-domain binding peptides, respectively, using the following formula:

$$PS(i,p)=\frac{n(i,p)}{N}$$

Where PS(*i*,*p*) is the position score of amino acid *i* at position *p*, n(*i*,*p*) is the number of sequences in which amino acid *i* occurs in position *p*, and N is the total number of peptide sequences in the respective dataset (i.e., 115 for SH3, 140 for WW, and 165 for PDZ). Perl scripts were written to calculate the positional scores for the 20 standard amino acids in each of the 6 positions for the SH3 & WW datasets, and 4 positions for the PDZ dataset, and these scores were used to generate PSSM scores for every query sequence by multiplying position scores for individual residues of the sequence.

### Regular Expression Scanning (RES) method

RES is a simple pattern matching algorithm implemented through Perl scripts, which involves mapping of the representative sequence patterns for positive examples of each domain-binding peptide class to the query sequences.

### Motif Instance Matching (MIM) method

MIM method relies on the alignment (mapping) of the query sequences with the peptide sequences in the non-redundant dataset collected from LMPID [17], for identifying exact matches that would result in positive prediction for a particular ligand class. Perl scripts were written to implement the MIM algorithm on each of the domain binding peptide classes.

### Cross-validation

To train, test and evaluate the classification models, we used the five-fold cross validation technique, in which, the whole dataset was divided into five sets such that in each round, four sets were used for training and the remaining one was set aside for testing. This process was repeated five times to ensure that each of the five sets was used once for testing and training.

### Performance measures

The performance of all the prediction methods was tested in terms of accuracy, sensitivity, specificity and Mathew’s Correlation Coefficient (MCC), using the following formulae:

$$Sensitivity=\frac{TP}{TP+FN}\times 100$$

$$Specificity=\frac{TN}{TN+FP}\times 100$$

$$Accuracy=\frac{TP+TN}{TP+FP+TN+FN}\times 100$$

$$MCC=\frac{\left(TP)(TN)-(FP)(FN\right)}{\sqrt{\left[TP+FP][TP+FN][TN+FP][TN+FN\right]}}\times 100$$

Where TP and TN are correctly predicted positive and negative examples, whereas, FP and FN are wrongly predicted positive and negative examples, respectively.

The models were also evaluated in a threshold independent manner by plotting receiver operating characteristic (ROC) curves and computing the respective area under the curve (AUC) values using ROCR package [33].

### Web implementation

The LMDIPred web server (*http://bicresources.jcbose.ac.in/ssaha4/lmdipred*) was developed using PHP 5.3.3, HTML and Perl scripts, and implemented using Apache HTTP 2.2.15 web server.

## Results

### Compositional analysis of different domain binding peptides

#### Amino acid composition

**Fig 1:**
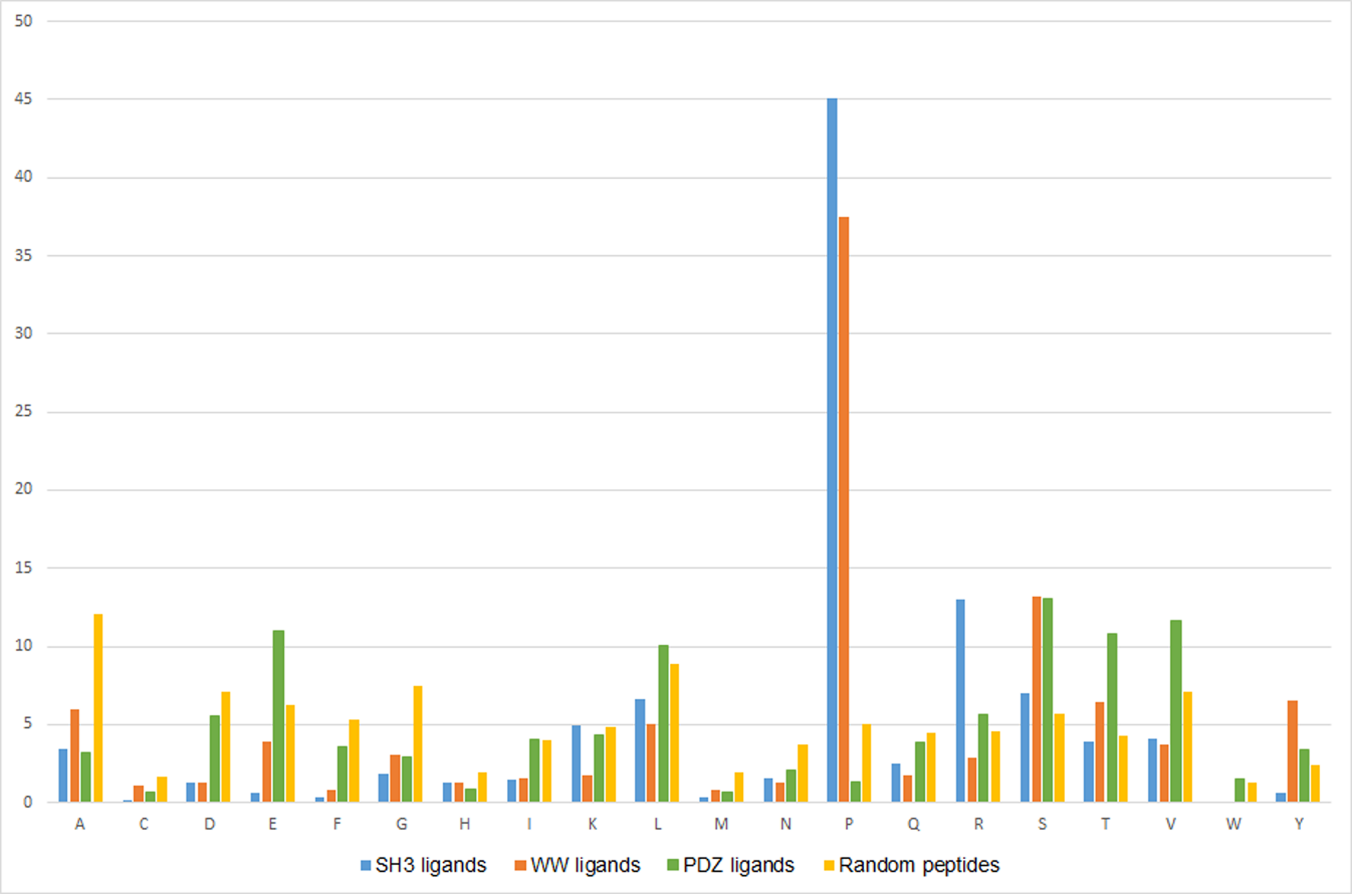
Bar graph depicting the average Amino Acid Composition (AAC) of SH3-binding, WW-binding, PDZ-binding and randomly generated peptide sequences.

We computed and compared the amino acid compositions of SH3, WW and PDZ ligand peptide sequences (**Fig 1**). In agreement with the existing knowledge, SH3 and WW ligand sequences were observed to be highly enriched in Proline (P) residues. SH3 ligands were found to contain a higher number of prolines than WW ligand, since there are two distinct xP dipeptide-binding pockets on the surface of SH3 domains as compared to a single such site on WW domains [26]. Also, SH3 ligands were enriched in Arginine (R) residues, whereas WW ligands contained more of Serine (S). PDZ ligand sequences were found to be enriched with amino acids like Serine (S), Threonine (T), Valine (V) and Glutamic acid (E). On performing ANOVA in IBM SPSS Statistics Version22.0, SH3 domain ligands were found to be significantly enriched (at 0.05 level) in Proline (P) and Arginine (R); WW domain ligands in Tyrosine (Y); and PDZ domain ligands in Glutamate (E), Threonine (T), and Valine (V).

#### Dipeptide composition

The dipeptide compositions of different classes of ligand peptide sequences were also computed and compared (**Table A** in **S1 File**). SH3 and WW domain-ligand sequences contained much higher number of diprolines (PP) and other Proline-containing dipeptides (xP or Px). PDZ domain ligands, on the other hand, were enriched in dipeptides containing Glutamate (xE or Ex) or Serine (xS or Sx).

#### Tripeptide composition

For each ligand class, we computed the tripeptide compositions, and on comparing the top 10 domain-specific tripeptides (**Table B** in **S1 File**), we found that for both SH3 and WW ligands, the tripeptides always contained Proline. Similarly, the top 10 PDZ ligands were found to be enriched in tripeptides containing either Glutamate or Serine.

### Support vector machine (SVM)-based models

In order to find the optimal peptide length for input data, we performed a pilot study using peptides of various lengths (4-mer, 6-mer, 8-mer, and 10-mer peptides) for each of the three domains. We observed that SVM models showed best performance measures with 6mer input for SH3 and WW binding peptides and 4-mer input for PDZ binders (**Fig B** and **Table D** in **S1 File**). Hence, we have developed prediction models using Support Vector Machine (SVM) for predicting 6-mer peptides that may bind to SH3 and WW, and 4-mer peptides to PDZ domains, and compared their prediction performances with different input features for the three different classes of peptide ligands (**Table 1**). The performance of SVM models were also found to be better than prediction models from other machine learning algorithms like Random Forest (RF) and Naïve Bayes (NB) classifiers (**Fig C** in **S1 File).**

**Table 1:** Comparison of prediction performance of SVM prediction models developed using different input features for different domain-binding peptides (6-mer peptides for SH3 and WW and 4-mer peptides for PDZ).

	AUC (%) values for different peptide classes		
	SH3-binding	WW-binding	PDZ-binding
Amino Acid Composition (AAC)	88.05	93.54	92.31
Dipeptide Composition (DPC)	86.79	96.33	93.65
Tripeptide Composition (TPC)	94.72	96.11	92.44
AAC + DPC	94.63	97.77	93.98
AAC + TPC	95.56	97.86	**97.69**
DPC + TPC	95.34	97.58	94.89
AAC + DPC + TPC	**97.45**	**98.35**	90.49

Based on these results, the SVM model developed using a combination of amino acid, dipeptide and tripeptide compositions (AAC+DPC+TPC) was selected for SH3 domain ligands, which achieved a maximum accuracy of 94.55% with MCC value of almost 0.85 on the unbalanced dataset (**Table 2**). The SVM model selected for WW domain ligands also used the same input feature combination i.e. AAC+DPC+TPC, and achieved the highest accuracy of 95.82% with MCC of nearly 0.90 on the unbalanced dataset (**Table 2**). For PDZ domain ligands, however, the SVM model using the combination of amino acid and tripeptide compositions (AAC+TPC) with C-terminal labelling was selected, giving the maximum accuracy of 92.29% with an MCC of 0.83 on the unbalanced dataset (**Table 2**). The prediction performances of the same models were also tested on the corresponding balanced datasets (**Table E (i) in S1 File**).

**Table 2:** Performance of SVM models for different domain binding peptides (6-mer peptides for SH3 and WW and 4-mer peptides for PDZ) on respective unbalanced datasets.

	P:N Ratio*	Threshold	Sensitivity	Specificity	Accuracy	MCC
**SH3**	~1:4	-0.25	0.9391	0.9471	0.9455	0.8475
**WW**	~1:3	-0.05	0.9571	0.9585	0.9582	0.8973
**PDZ**	~1:2	-0.10	0.9152	0.9263	0.9229	0.8259

*P:N Ratio denotes ratio of positive to negative data

### Position-Specific Scoring Matrix (PSSM) scanning

Position-Specific Scoring Matrices (PSSMs) were constructed for SH3, WW and PDZ ligands, using alignments of the 115 SH3-domain binding peptides, 140 WW-domain binding peptides and 165 PDZ-domain binding peptides described above. The PSSM-scanning method was evaluated using the five-fold cross-validation technique on both the balanced and unbalanced datasets for each ligand class. We obtained a maximum accuracy of 87.82% (MCC 0.62) for SH3 ligands, 85.09% (MCC 0.66) for WW ligands, and 86.36% (MCC 0.68) for PDZ ligands for this method on the respective unbalanced datasets (**Table 3**).

**Table 3:** Performance of PSSMs for different domain binding peptides (6-mer peptides for SH3 and WW and 4-mer peptides for PDZ) on respective unbalanced datasets.

	P:N Ratio	Threshold	Sensitivity	Specificity	Accuracy	MCC
**SH3**	~1:4	1.00	0.6957	0.9264	0.8782	0.6167
**WW**	~1:3	0.50	0.8786	0.8415	0.8509	0.6640
**PDZ**	~1:2	0.60	0.6857	0.9467	0.8636	0.6774

### Regular Expression Scanning (RES)

The linear motif expressions that have been found to represent peptide sequences binding to SH3, WW and PDZ domains in experimental studies, were compiled into domain-specific lists, and 6-mer query sequences were scanned for their presence using regular expression mapping Perl programs. Five-fold cross-validation of this method yielded a maximum accuracy of 89.09% (MCC 0.67) for SH3 ligands, 96.55% (MCC 0.91) for WW ligands, and 83.45% (MCC 0.63) for PDZ ligands on the respective unbalanced datasets (**Table 4**).

**Table 4:** Performance of RES method for different domain binding peptide classes on respective unbalanced datasets.

	P:N Ratio	Sensitivity	Specificity	Accuracy	MCC
**SH3**	~1:4	0.8087	0.9126	0.8909	0.6735
**WW**	~1:3	0.8929	0.9902	0.9655	0.9064
**PDZ**	~1:2	0.7657	0.8667	0.8345	0.6305

### Motif Instance Matching (MIM)

The experimentally validated linear motif instances reported in scientific literature to bind to SH3, WW and PDZ domains were collected from the LMPID database and matched to 6-mer query sequences using exact string-matching programs written in Perl. This method was also evaluated on the unbalanced datasets using five-fold cross-validation, producing a maximum accuracy of 82.73% (MCC 0.36) for SH3 ligands, 77.82% (MCC 0.29) for WW ligands, and 77.82% (MCC 0.46) for PDZ ligands (**Table 5**). The performance measures reflected very low sensitivity (17.39% for SH3, 12.86% for WW and 30.29% for PDZ ligands), but high specificity values (100% for all three) for the three ligand classes. It is an expected outcome of this method, since it can only search for already known motif sequences, but cannot identify novel sequences differing by even a single residue from the known motif instances. However, this method may be used by users who might want to restrict the occurrence of false positives in their prediction results.

**Table 5:** Performance of MIM method for different domain binding peptide classes on respective unbalanced datasets.

	P:N Ratio	Sensitivity	Specificity	Accuracy	MCC
**SH3**	~1:4	0.1739	1.0000	0.8273	0.3642
**WW**	~1:3	0.1286	1.0000	0.7782	0.2915
**PDZ**	~1:2	0.3029	1.0000	0.7782	0.4655

### Performance comparison of different prediction methods

**Fig 2:**
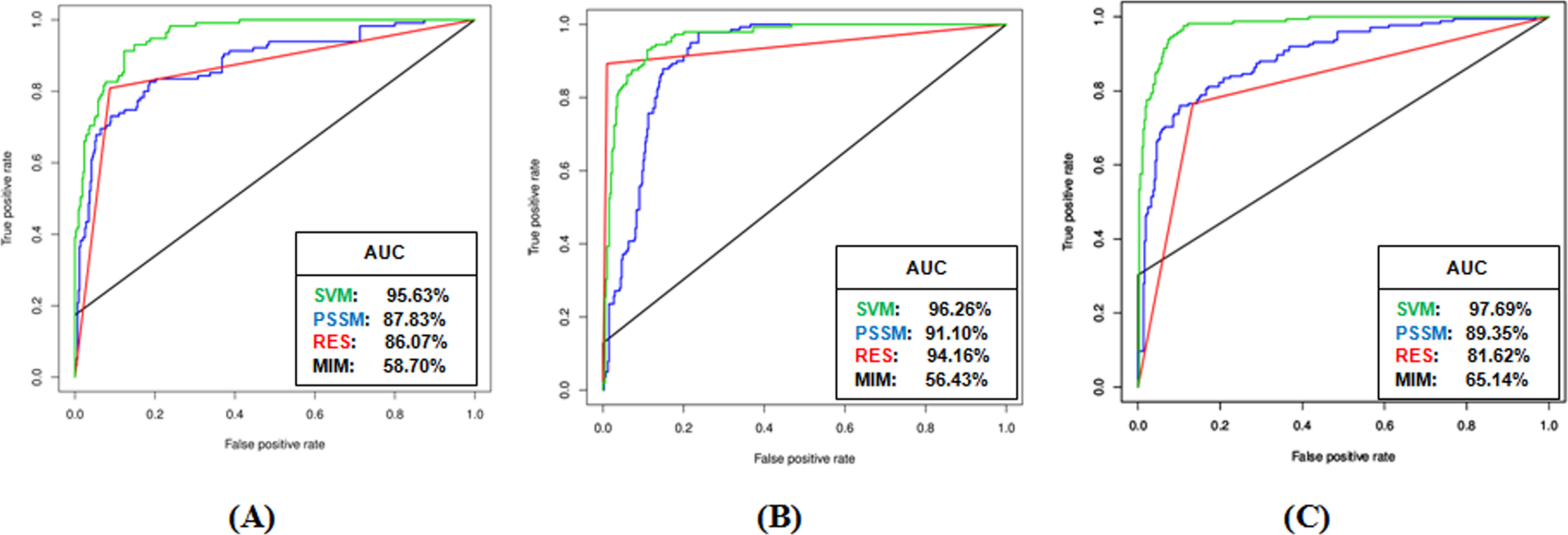
**ROC plots showing prediction performances of the different methods (Green-SVM, Blue-PSSM, Red- Regular Expression Scanning (RES), Black- Motif Instance Matching (MIM)) for (A) SH3 domain ligands, (B) WW domain ligands, and (C) PDZ domain ligands.** The respective AUC values are mentioned in the corresponding textboxes.

We have compared the threshold independent performance of the different prediction methods during five-fold cross-validation on the domain-specific unbalanced datasets described above, by plotting receiver-operating-characteristic (ROC) curves (**Fig 2**) and computing the respective area-under-the-curve (AUC). Based on the ROC plots, SVM models for all three domains were found to outperform the other prediction methods, while, Motif Instance Matching method performed poorly for all domains.

### Performance of prediction methods on independent datasets

All the above methods were evaluated using two independent datasets not used for training or testing, which was composed of 62 experimentally validated PDZ-binding 10-mer mouse peptides [29] and 25 experimentally validated SH3-binding yeast peptides of variable length [30] (**Table 6**). The sensitivity values of SVM and PSSM scanning methods were reported at the threshold values which gave the best accuracy during five-fold cross-validation. Thereafter, all the four methods were integrated to create the LMDIPred web server, so that users can be provided with a comprehensive prediction result.

**Table 6:** Comparison of sensitivity shown by different prediction methods on the independent datasets.

	Sensitivity (%) for SH3 ligands	Sensitivity (%) for PDZ ligands
SVM	60.00	75.81
PSSM	32.00	69.35
RES	80.00	93.55
MIM	16.00	29.03
LMDIPred (Combined)*	92.00	97.00
MoDPepInt	40.00	100.00
ELM	84.00	53.23

*Hits from any one of the four methods.

We have also compared the union of predictions from all four methods in LMDIPred on the independent datasets, with that of SH3PepInt [15] and PDZPepInt [16] utilities available in the MoDPepInt web server [13] (**Table 6**), as well as the motif prediction method provided by the ELM database [9]. Out of the 25 experimentally validated SH3-binding peptide sequences, LMDIPred could correctly predict 23, whereas, SH3PepInt could identify only 10. However, among the 62 bonafide PDZ domain ligand peptides, LMDIPred could identify 60, while PDZPepInt could detect all 62 sequences. This result proves that predictions from LMDIPred are reliable, and performance of this web server is comparable to the existing ones.

### LMDIPred web server

**Fig 3:**
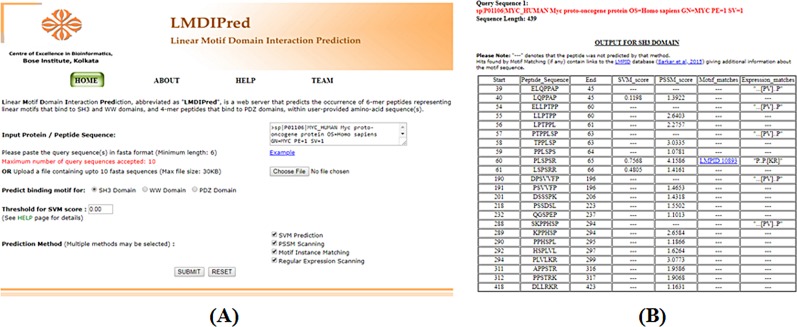
(A) Screenshot of the home page of LMDIPred with sample input. (B) Screenshot of a sample output from LMDIPred web server.

The principal aim of this study was to develop a publicly available online platform that can be used to predict the occurrence of possible peptide ligands to SH3, WW or PDZ domains, within a user-provided amino-acid sequence. To fulfil this objective, we have developed the LMDIPred web server, available at *http://bicresources.jcbose.ac.in/ssaha4/lmdipred*. LMDIPred allows its users to submit up to ten fasta-formatted protein or peptide sequences containing 6 or more residues, as input, either by pasting directly into the text-area provided for this purpose (**Fig 3A**), or by uploading a '.txt' or '.fasta' sequence file. Any one, two, three or all four of the prediction methods, viz., (i) SVM Prediction, (ii) PSSM Scanning, (iii) Motif Instance Matching, and, (iv) Regular Expression Scanning, may be selected for predicting ligand peptides to any one of the domains. The threshold score for SVM prediction can also be set by the user (default value 0.00, i.e., any contiguous stretch of amino acids with a positive SVM prediction score will be predicted to bind to the chosen domain). Higher threshold values make the search more stringent, resulting in higher specificity but lower sensitivity, thus missing some of the genuine motifs. Lowering the threshold, on the other hand, may increase sensitivity but will decrease specificity, thereby producing spurious hits. All the input options and parameters have been discussed in detail in the ‘Help’ page of LMDIPred for the benefit of its users.

The LMDIPred server provides a combined output result in a tabular format (**Fig 3B**), which represents a union of the prediction results from the four independent methods, for each of the user-provided query sequences, one after another. The output table for each query displays the peptide hits from one or more method(s) according to their sequence positions, alongside the outputs from all the four methods. SVM and PSSM scores are provided for the peptides predicted by the corresponding SVM models and PSSMs, respectively. The peptides predicted by Motif Instance Matching are linked to corresponding entries in the LMPID database, to provide information about the matching LM instance, like its critical residues, post-translational modifications, secondary structure and experimental methods used to validate it. For peptides predicted by Regular Expression Scanning, the matching regular expressions are mentioned in the output. A ´—´ in the output column of any method, against a particular peptide in the output table, denotes that the peptide hit was not present in the predictions from that particular method. Prediction of the same peptide region by three or more methods strongly indicates the presence of a peptide ligand to the corresponding domain.

## Discussion

Accurate computational methods for prediction of peptide-mediated PPIs, may augment experimental studies on these PPIs, and thereby help in elucidating their role in complex regulatory pathways, as well as, provide opportunities for discovery of novel therapeutic modulators. The highly promiscuous binding patterns displayed by the peptide-binding domains, reflecting their intrinsic ability to recognize a diverse set of ligands, makes the prediction of specific domain-binding peptides a highly challenging task. In this scenario, we have made an attempt to develop user-friendly computational methods to predict novel peptide sequences that may mediate protein interactions by binding to specific peptide-binding domains like SH3, WW and PDZ.

We have compiled the positive training datasets comprising of experimentally validated SH3, WW, and PDZ domain binding peptides from the LMPID database [17]. Random peptides from food proteins as well as peptides from the positive set of the other two domains served as the negative dataset for ligands of each domain. For example, the negative dataset for SH3 ligand class consisted of the positive dataset for WW and PDZ ligand classes as well as randomly generated food peptides. Inclusion of peptide ligands of other domains in the negative dataset ensured that the prediction models would be able to distinguish true domain binding peptides from LM-containing non-binding peptides. We observed that the best prediction performance for SVM models was achieved on using 6-mer peptides as input for SH3 and WW domain binders and 4-mer peptides for PDZ domain ligands.

During five-fold cross-validation studies, we found that the machine learning models performed better than sequence-matching approaches in predicting ligands for different domains. This performance is presumably because SVM models were more capable of assigning the residues preferred in the wildcard positions (denoted by x, as in xxPxxP motif binding to SH3 domains), based on statistically computed bias for each residue in each position, which is derived from training data. Thus, the ROC plots showed that the prediction performance of SVM was the best among all the prediction methods, while that of Motif Instance Matching method was by far the worst, due to poor sensitivity. It is observed that the ROC plots for MIM and RES methods appear as smooth flat lines when compared to the plots for SVM and PSSM, because SVM and PSSM outputs comprise of continuous scores, while the MIM and RES produce discrete outcomes, one or zero (either “match” or “mismatch”). We also observed that, though, AUC values indicate almost similar performances for PSSM and RES methods during five-fold cross-validation, RES showed much better performance than PSSM on independent datasets. It should also be noted that RES method performed better than the other methods on the independent datasets, because we have only measured sensitivity (the percent of correctly predicted ligands), which is expected to be high for RES since it allows greater flexibility in the wildcard positions than the other methods. Nevertheless, all four methods have been included in LMDIPred web server to provide a combined prediction output, from which predicted domain-binding peptides may be picked by the users. The combined prediction results of LMDIPred server were also compared with that of the MoDPepInt server and ELM, and the performance of our web server was found to be appreciable.

Overall, the LMDIPred web server is an attempt to provide a preliminary platform for in-silico prediction of peptide sequences that may interact with SH3, WW or PDZ domains, to facilitate experimental studies that may lead to discovery and characterization of novel PPIs. Furthermore, we have provided the datasets used in the present study on our web server to help the scientific community in developing better methods for prediction of such domain binding peptides.

## Supporting information

S1 File This is the S1 File description. Fig A: Distribution of motif lengths among SH3, WW and PDZ binding peptides.
Fig B: ROC plots for SVM classifiers of (i) SH3, (ii) WW and (iii) PDZ binding peptides using 4-mer (Blue), 6-mer (Green), 8-mer (Black) and 10-mer (Red) peptides as input.
Fig C: Comparison between prediction accuracy of Support Vector Machine (SVM), Random Forest (RF) and Naïve Bayes (NB) classifiers for SH3 (6-mer), WW (6-mer) and PDZ (4-mer) binding peptides.
Table A (i): Distribution of the most abundant (top 10) dipeptides found in SH3 domain-ligands among different peptide classes (n denotes total number of peptides in each class).
Table A (ii): Distribution of the most abundant (top 10) dipeptides found in WW domain-ligands among different peptide classes (n denotes total number of peptides in each class).
Table A (iii): Distribution of the most abundant (top 10) dipeptides found in PDZ domain-ligands among different peptide classes (where n denotes total number of peptides in each class).
Table B (i): Distribution of the most abundant (top 10) tripeptides found in SH3 domain-ligands among different peptide classes (where n denotes total number of peptides in each class).
Table B (ii): Distribution of the most abundant (top 10) tripeptides found in WW domain-ligands among different peptide classes (where n denotes total number of peptides in each class).
Table B (iii): Distribution of the most abundant (top 10) tripeptides found in PDZ domain-ligands among different peptide classes (where n denotes total number of peptides in each class).
Table C: Optimized parameters of SVM classification models for different domain binding peptide classes.
Table D: Performance of SVM classification models for varying peptide lengths of different domain binding peptide classes.
Table E (i): Performance of SVM models for different domain binding peptide classes on respective Balanced Datasets.
Table E (ii): Performance of PSSMs for different domain binding peptide classes on respective Balanced Datasets.
Table E (iii): Performance of RES method for different domain binding peptide classes on respective Balanced Datasets.
Table E (iv): Performance of MIM method for different domain binding peptide classes on respective Balanced Datasets.
(PDF)
