# Supplementary material for: LMDIPred: A web-server for prediction of linear peptide sequences binding to SH3, WW and PDZ domains
Source: PLoS One. 2018 Jul 12;13(7):e0200430. doi: 10.1371/journal.pone.0200430 (PMC6042728; doi:10.1371/journal.pone.0200430)
Supplement: S1 File — Fig A: Distribution of motif lengths among SH3, WW and PDZ binding peptides. Fig B: ROC plots for SVM classifiers of (i) SH3, (ii) WW and (iii) PDZ binding peptides using 4-mer (Blue), 6-mer (Green), 8-mer (Black) and 10-mer (Red) peptides as input. Fig C: Comparison between prediction accuracy of Support Vector Machine (SVM), Random Forest (RF) and Naïve Bayes (NB) classifiers for SH3 (6-mer), WW (6-mer) and PDZ (4-mer) binding peptides. Table A (i): Distribution of the most abundant (top 10) dipeptides found in SH3 domain-ligands among different peptide classes (n denotes total number of peptides in each class). Table A (ii): Distribution of the most abundant (top 10) dipeptides found in WW domain-ligands among different peptide classes (n denotes total number of peptides in each class). Table A (iii): Distribution of the most abundant (top 10) dipeptides found in PDZ domain-ligands among different peptide classes (where n denotes total number of peptides in each class). Table B (i): Distribution of the most abundant (top 10) tripeptides found in SH3 domain-ligands among different peptide classes (where n denotes total number of peptides in each class). Table B (ii): Distribution of the most abundant (top 10) tripeptides found in WW domain-ligands among different peptide classes (where n denotes total number of peptides in each class). Table B (iii): Distribution of the most abundant (top 10) tripeptides found in PDZ domain-ligands among different peptide classes (where n denotes total number of peptides in each class). Table C: Optimized parameters of SVM classification models for different domain binding peptide classes. Table D: Performance of SVM classification models for varying peptide lengths of different domain binding peptide classes. Table E (i): Performance of SVM models for different domain binding peptide classes on respective Balanced Datasets. Table E (ii): Performance of PSSMs for different domain binding peptide classes on respective Balanced Data [file pone.0200430.s001.pdf]

LMDIPred: A web-server for prediction of linear peptide sequences binding to SH3, WW and PDZ domains.

Debasree Sarkar[1], Tanmoy Jana[1], and Sudipto Saha[1]*

[1] Bioinformatics centre, Bose Institute, Kolkata, India.

Supplementary Information

Supplementary Figures:

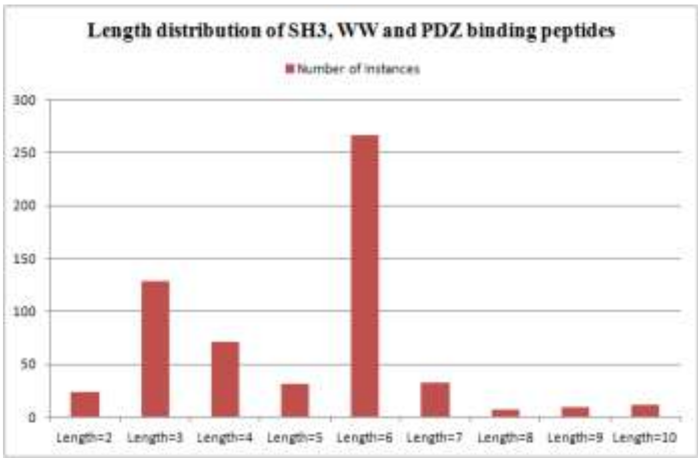

Fig A: Distribution of motif lengths among SH3, WW and PDZ binding peptides.

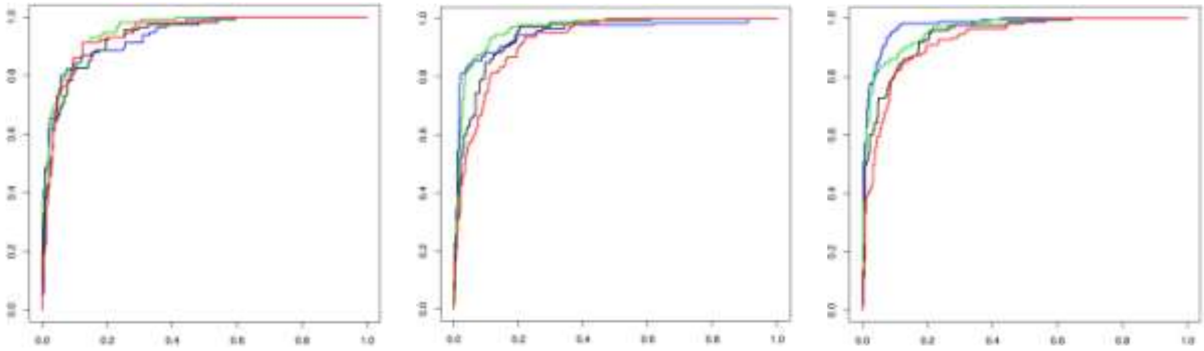

(i) (ii) (iii)

Fig B: ROC plots for SVM classifiers of (i) SH3, (ii) WW and (iii) PDZ binding peptides using 4-mer (Blue), 6-mer (Green), 8-mer (Black) and 10-mer (Red) peptides as input.

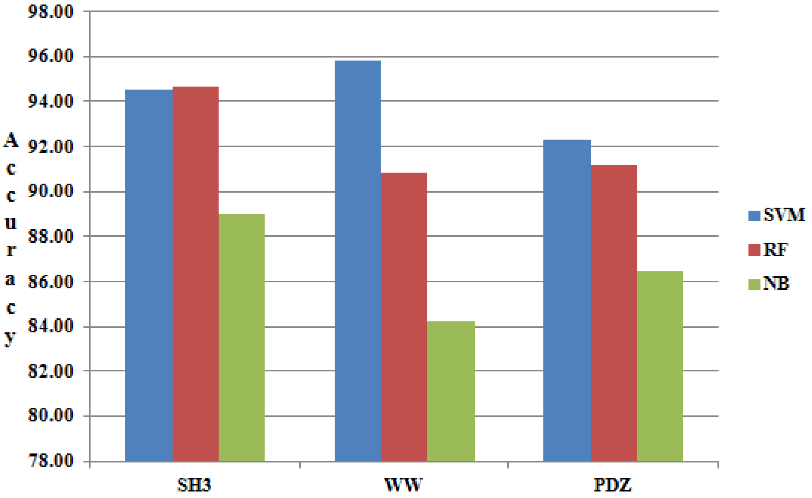

Fig C: Comparison between prediction accuracy of Support Vector Machine (SVM), Random Forest (RF) and Naïve Bayes (NB) classifiers for SH3 (6-mer), WW (6-mer) and PDZ (4-mer) binding peptides.

Supplementary Tables:

Table A (i): Distribution of the most abundant (top 10) dipeptides found in SH3 domain-ligands among different peptide classes (n denotes total number of peptides in each class):

	**SH3** (n=115)	**WW** (n=140)	**PDZ** (n=165)	**Random** (n=120)
PP	82	113	0	3
LP	23	9	0	2
PS	21	13	0	1
PL	18	8	0	2
RP	18	1	2	1
PA	17	4	4	2
PV	17	4	3	1
VP	14	2	2	1
AP	11	8	0	5
KP	11	1	0	0

Table A (ii): Distribution of the most abundant (top 10) dipeptides found in WW domain-ligands among different peptide classes (n denotes total number of peptides in each class):

	**WW** (n=140)	**SH3** (n=115)	**PDZ** (n=165)	**Random** (n=120)
PP	113	82	0	3
SP	66	9	0	0
TP	31	6	1	0
PY	27	1	0	0
PS	13	21	0	1
LS	11	2	1	5
LP	9	23	0	2
SS	9	4	17	4
AP	8	11	0	5
PL	8	18	0	2

Table A (iii): Distribution of the most abundant (top 10) dipeptides found in PDZ domain-ligands among different peptide classes (where n denotes total number of peptides in each class):

	**PDZ** (n=165)	**SH3** (n=115)	**WW** (n=140)	**Random** (n=120)
ES	28	0	2	3
ET	27	0	2	1
RE	18	1	0	0
SS	17	4	9	4
LE	14	0	0	3
RR	14	0	0	1
SD	13	0	1	2
SL	13	1	1	2
SV	13	0	0	1
TE	13	0	0	1

Table B (i): Distribution of the most abundant (top 10) tripeptides found in SH3 domain-ligands among different peptide classes (where n denotes total number of peptides in each class):

	**SH3** (n=115)	**WW** (n=140)	**PDZ** (n=165)	**Random** (n=120)
PPP	23	57	0	2
PPR	21	3	0	1
PLP	14	4	0	0
PPK	12	1	0	0
PVP	11	0	1	0
LPP	10	5	0	0
PPV	10	2	0	0
PSR	10	0	0	0
VPP	10	2	0	0
PRP	9	0	0	0

Table B (ii): Distribution of the most abundant (top 10) tripeptides found in WW domain-ligands among different peptide classes (where n denotes total number of peptides in each class):

	**WW** (n=140)	**SH3** (n=115)	**PDZ** (n=165)	**Random** (n=120)
PPP	26	23	0	2
PPY	26	1	0	0
SPP	14	0	0	0
LSP	10	2	0	0
SPS	9	3	0	0
SSP	8	0	0	0
TPP	8	4	0	0
PPL	6	4	0	0
PPS	6	1	0	0
SPR	6	2	0	0

Table B (iii): Distribution of the most abundant (top 10) tripeptides found in PDZ domain-ligands among different peptide classes (where n denotes total number of peptides in each class):

	**PDZ** (n=165)	**SH3** (n=115)	**WW** (n=140)	**Random** (n=120)
RET	12	0	0	0
RRE	11	0	0	0
TEV	9	0	0	0
ESE	8	0	0	0
IES	8	0	0	0
LES	8	0	0	1
SDV	8	0	0	0
SLE	8	0	0	0
SSV	8	0	0	0
EFY	7	0	0	0

Table C: Optimized parameters of SVM classification models for different domain binding peptide classes:

	Kernel Function	*Error Penalty (C)*	*Free parameter (Gamma)*
**SH3 domain**	RBF	2	0.0005
**WW domain**	RBF	2	0.0005
**PDZ domain**	RBF	1	0.0005

Table D: Performance of SVM classification models for varying peptide lengths of different domain binding peptide classes:

Domain	Peptide Length	Sensitivity	Specificity	Accuracy	AUC
SH3	4mer	83.48	93.02	91.01	93.71
	**6mer**	**93.91**	**94.88**	**94.55**	**95.24**
	8mer	76.52	94.65	90.83	93.54
	10mer	83.48	94.19	91.93	94.41
WW	4mer	84.14	94.75	91.93	95.02
	**6mer**	**95.71**	**95.85**	**95.82**	**97.32**
	8mer	84.83	93.75	91.38	94.27
	10mer	77.24	92.75	88.62	92.45
PDZ	**4mer**	**93.94**	**92.11**	**92.66**	**97.64**
	6mer	88.00	91.47	90.36	96.03
	8mer	84.24	90.53	88.62	94.16
	10mer	86.67	89.21	88.44	92.75

Table E (i): Performance of SVM models for different domain binding peptide classes on respective Balanced Datasets:

	P:N Ratio	Threshold	Sensitivity	Specificity	Accuracy	MCC
**SH3**	1:1	0.00	0.9565	0.8870	0.9217	0.8543
**WW**	1:1	0.00	0.9857	0.8857	0.9357	0.8787
**PDZ**	1:1	0.05	0.8914	0.9086	0.9000	0.8058

Table E (ii): Performance of PSSMs for different domain binding peptide classes on respective Balanced Datasets:

	P:N Ratio	Threshold	Sensitivity	Specificity	Accuracy	MCC
**SH3**	1:1	0.50	0.7217	0.9652	0.8435	0.7174
**WW**	1:1	0.50	0.8786	0.8429	0.8607	0.7252
**PDZ**	1:1	0.00	0.7600	0.9486	0.8543	0.7312

Table E (iii): Performance of RES method for different domain binding peptide classes on respective Balanced Datasets:

	P:N Ratio	Sensitivity	Specificity	Accuracy	MCC
**SH3**	1:1	0.8087	0.9739	0.8913	0.8006
**WW**	1:1	0.8929	0.9857	0.9393	0.8908
**PDZ**	1:1	0.7657	0.9029	0.8343	0.6950

Table E (iv): Performance of MIM method for different domain binding peptide classes on respective Balanced Datasets:

	P:N Ratio	Sensitivity	Specificity	Accuracy	MCC
**SH3**	1:1	0.1739	1.0000	0.5870	0.2989
**WW**	1:1	0.1286	1.0000	0.5643	0.2445
**PDZ**	1:1	0.3029	1.0000	0.6514	0.4143
